# Eicosapentaenoic acid shows anti-inflammatory effect via GPR120 in 3T3-L1 adipocytes and attenuates adipose tissue inflammation in diet-induced obese mice

**DOI:** 10.1186/s12986-017-0188-0

**Published:** 2017-05-08

**Authors:** Hodaka Yamada, Tomio Umemoto, Masafumi Kakei, Shin-ichi Momomura, Masanobu Kawakami, San-e Ishikawa, Kazuo Hara

**Affiliations:** 1 First Department of Comprehensive Medicine, Jichi Medical University Saitama Medical Center, 1-847 Amanumacho, Omiya, Saitama 330-8503 Japan; 2 Internal Medicine, Nerima Hikarigaoka Hospital, 2-11-1 Hikarigaoka, Nerima, Tokyo, 179-0072 Japan; 3 Division of Endocrinology and Metabolism, International University of Health and Welfare Hospital, 537-3 Iguchi, Nasushiobara, Tochigi 329-2763 Japan

**Keywords:** Palmitate, Adipocytes, G-protein coupled receptor 120, Eicosapentaenoic acid, Macrophage phenotype

## Abstract

**Background:**

Saturated fatty acids have been shown to cause insulin resistance and low-grade chronic inflammation, whereas unsaturated fatty acids suppress inflammation via G-protein coupled receptor 120 (GPR120) in macrophages. However, the anti-inflammatory effects of unsaturated fatty acids in adipocytes have yet to be elucidated. Hence, the aims of the present study were to evaluate the anti-inflammatory effects of eicosapentaenoic acid (EPA) via GPR120 in adipocytes.

**Methods:**

We used 250 μM palmitate as a representative saturated fatty acid. 3T3-L1 adipocytes were used for in vitro studies. We further evaluated the effect of EPA supplementation in a high-fat/high-sucrose (HFHS) diet-induced adipose tissue inflammatory mouse model.

**Results:**

EPA attenuated palmitate-induced increases in inflammatory gene expression and NF-κB phosphorylation in 3T3-L1 adipocytes. Silencing of GPR120 abolished the anti-inflammatory effects of EPA. In GPR120 downstream signal analysis, EPA was found to decrease palmitate-induced increases in TAK1/TAB1 complex expression. EPA supplementation suppressed HFHS-induced crown-like structure formation in epididymal adipose tissue and altered macrophage phenotypes from M1 to M2 in the stromal vascular fraction. Moreover, the EPA-containing diet attenuated increases in adipose p-JNK and phospho-p65 NF-κB levels.

**Conclusions:**

In conclusion, the findings of the present study demonstrate that EPA suppresses palmitate-induced inflammation via GPR120 by inhibiting the TAK1/TAB1 interaction in adipocytes. EPA supplementation reduced HFHS diet-induced inflammatory changes in mouse adipose tissues. These results demonstrate adipose GPR120 as a potential therapeutic target for decreasing inflammation.

**Electronic supplementary material:**

The online version of this article (doi:10.1186/s12986-017-0188-0) contains supplementary material, which is available to authorized users.

## Background

Obesity is known to contribute to the pathogenesis of metabolic syndrome and established risk factor for cardiovascular disease [1]. Obesity causes insulin resistance due to chronic low-grade inflammation [2, 3]. In obese states, plasma free fatty acids (FFAs) levels, particularly saturated free fatty acids (SFAs) such as palmitate, are elevated and contribute to lipotoxicity in various organs [4]. On the other hand, polyunsaturated free fatty acids (PUFAs) such as eicosapentaenoic acid (EPA), an omega-3 (ω-3) fatty acid, have been shown to have anti-inflammatory effects. Indeed, the long-term use of EPA supplementation in hypercholesterolemic Japanese patients reduced the risk of major coronary events [5]. Accordingly, FFAs are considered to have dual activity, namely inflammatory and anti-inflammatory effects. Recently, Oh and Olefsky et al. identified G-protein coupled receptor 120 (GPR120, Free Fatty Acid Receptor 4: FFAR4) as a receptor for ω-3 fatty acids and demonstrated improved obesity-induced insulin resistance via GPR120 signaling in macrophages [6]. Oh et al. demonstrated that docosanexaenoic acid (DHA), a ω-3 fatty acid, inhibits downstream nuclear factor-κB (NF-κB) signaling via tumor necrosis factor-α (TNF-α) receptor and toll-like receptor 4 (TLR4) activation in response to lipopolysaccharide (LPS) in macrophages [6, 7]. GPR120 is known to be expressed in adipose and intestinal tissues, in addition to macrophages [6, 8]. Intestinal GPR120 activation increases secretion of glucagon-like peptide-1 and has been shown to have an anti-diabetic effect [8]. However, the mechanisms underlying anti-inflammatory effects of EPA treatment in adipocytes have yet to be elucidated. In the present study, we hypothesized that EPA directly affects adipocytes inflammation via GPR120 and ameliorates diet-induced obesity. The aims of the present study were to determine the anti-inflammatory effects of EPA via GPR120 in cultured adipocytes, and to clarify the effects of EPA supplementation on high-fat/high-sucrose (HFHS) diet-induced inflammatory changes in adipose tissue.

## Methods

### Reagents

Palmitate (Sigma–Aldrich) was used after conjugation with fatty acid-free albumin (Sigma–Aldrich). EPA sodium salt (EPA–Na) was purchased from Nu-Chek Prep, Inc. for cell treatment. Culture medium containing EPA was prepared according to previously described methods [9]. Highly purified EPA for mice was kindly provided by Mochida Pharmaceutical Company, Ltd.

### Cell culture, treatment, and harvesting

3T3-L1 murine pre-adipocytes were purchased from American Type Tissue Culture Collection and differentiated according to a previously described standard protocol. [10] Fully differentiated 3T3-L1 adipocytes were exposed to 250 μM palmitate for 30 min, 60 min, or 24 h with or without a 6-h pretreatment with 50 μM EPA-Na. Cells were harvested by RIPA buffer with phosphatase and protease inhibitors (nacalai tesque) for protein extraction.

### Animal studies

Male C57BL/6J mice (CLEA Japan, Inc.) were housed in accordance with our institutional guidelines and the Japanese Physiological Society’s guidelines for animal care in an air-conditioned room with a 12-h light/dark cycle, with food and water available ad libitum. Mice were divided into three groups and fed a standard chow diet (MF diet, ORIENTAL YEAS CO.,LTD), high fat/high sucrose (HFHS) diet (30% fat, 20% sucrose) or HFHS diet supplemented with EPA (5% wt/wt) for 24 weeks from 4 weeks of age. At the end of the study, mice were anesthetized by the intraperitoneal injection of pentobarbital (100 mg/kg) and then sacrificed. All experimental protocols for animal studies were approved by the institutional committee on animal care at Jichi Medical University.

### Plasma analysis

Plasma insulin and adiponectin levels were measured by mouse insulin ELISA kits (Morinaga) and by adiponectin Bio-Plex Pro (BIO RAD), respectively. We performed blood glucose measurements at 12 and 18 weeks (Terumo Medisafe Mini Glucometer). Other plasma measurements were performed at Nagahama Life Science Laboratory (Shiga, Japan). The homeostasis model for insulin resistance (HOMA-IR) was calculated using the following formula: HOMA-IR = fasting plasma glucose (mg/dL) × fasting plasma insulin/405.

### Stromal vascular fraction isolation

The stromal vascular (SV) fraction was isolated from epididymal adipose tissue according to previously described methods with some modifications [11, 12]. In brief, after sacrifice, epididymal adipose tissues were resected, minced (<2 mm), and digested with collagenase (Sigma-Aldrich) in Krebs-Henseleit-HEPES buffer, pH 7.4, containing 20 mg/mL of BSA and 2.8 mM glucose at 37 °C using a shaker for 45 min. Then, samples were passed through a 40-μm mesh and centrifuged (1,000 g for 8 min). Pellets and floating cells were collected as the SV fraction and primary adipocytes, respectively. Isolated cells were then used for RNA extraction.

### Transfection and Real-time quantitative reverse-transcription polymerase chain reaction

RNA was isolated from 3T3-L1 adipocytes and primary adipocytes with Direct-zol™ RNA MiniPrep (Zymo Research). Complementary DNA was made with ReverTra Ace qPCR RT Master Mix (TOYOBO). Reverse transcription polymerase chain reaction (RT-PCR) was performed using TaqMan® gene expression assays (Applied Biosystems). Primer-probe sets are shown in Additional file 1: Table S1. All mRNA levels were normalized by GAPDH, as an internal control gene. Relative gene expression levels were calculated using the ΔΔCT method as previously described [13]. Gene silencing of *GPR120* was performed using mouse *GPR120* siRNA (Santa cruz) according to the manufacturer’s protocol. The siRNA efficiency was determined using western blotting.

### Western blot analysis and co-immunoprecipitation

Total cell lysates were obtained as previously described [10] and nuclear protein fractions were extracted using Universal Magnetic Co-IP kits (Active Motif) according to the manufacturer’s protocol. Protein samples were resuspended in SDS sample buffer and boiled at 100 °C for 3 min. The same volume of each sample was applied and separated by SDS-PAGE and electrophoretically transferred to polyvinylidene difluoride membranes. Membranes were immunoblotted using the following primary antibodies: tumor growth factor β (TGF-β) activated kinase 1 (TAK1), TGF-β activated kinase 1 binding protein 1 (TAB1) (Santa Cruz Biotechnology, SC-6052, lot: C1114), phospho-interferon regulatory factor 3 (IRF3) (Cell signaling, #4947, lot: 7), total c-Jun NH2-terminal kinase (JNK) (Cell signaling, #9252, lot: 15), phospho-JNK (Cell signaling, #9251, lot: 21), NF-κB-p65 (Cell signaling, #8242, lot: 4), and phospho-NF-κB-p65 (Cell signaling, #3031, lot: 8), TNF receptor-associated factor 6 (TRAF6) (Invitrogen, 38–0900, lot: 1308635A), GPR120 (Abcam, ab97272, lot: GP26308-24). Co-immunoprecipitation to evaluate the interaction between TAK1 and TAB1 was performed using Universal Magnetic Co-IP kits (Active Motif) according to the manufacturer’s protocol. Collected lysates and immune complexes were analyzed by western blotting. All signals were analyzed by C-Digit (LI-COR). GAPDH antibodies (Wako) were used as an internal control.

### Histological analyses

Macrophage infiltration into epididymal adipose and hepatic tissues was evaluated using MAC-2 immunostaining. MAC-2 antibody staining and hematoxylin and eosin (HE) staining were performed at Biopathology Institute Co,. Ltd. (Oita, Japan). The number of crown-like structures (CLSs) was counted in five independent fields and recorded as the mean number of CLDs per low-power field (Olympus BX-51, ×100). Adipocyte diameter was measured as the mean diameter of 100 adipocytes in each slide.

### Statistical analysis

All data are shown as means ± SEM. Statistical significance was determined using the unpaired Student's *t*-test. Spearman’s correlation was used for linear regression analysis. Prism version 5 (GraphPad software Inc.) was used for statistical analyses. *P*-values of <0.05 were considered statistically significant.

## Results

**Fig. 1:**
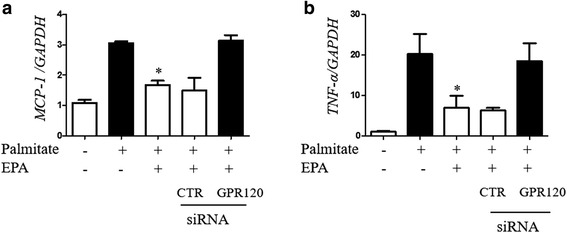
mRNA expression of palmitate-induced MCP-1 (**a**) and TNF-α (**b**) in 3T3-L1 adipocytes with or without GPR120 silencing. Before palmitate exposure, EPA pretreatment was performed. Data are presented as the mean ± SEM of three independent experiments. *P < 0.05 vs. palmitate exposure. CTR; scramble siRNA

**Fig. 2:**
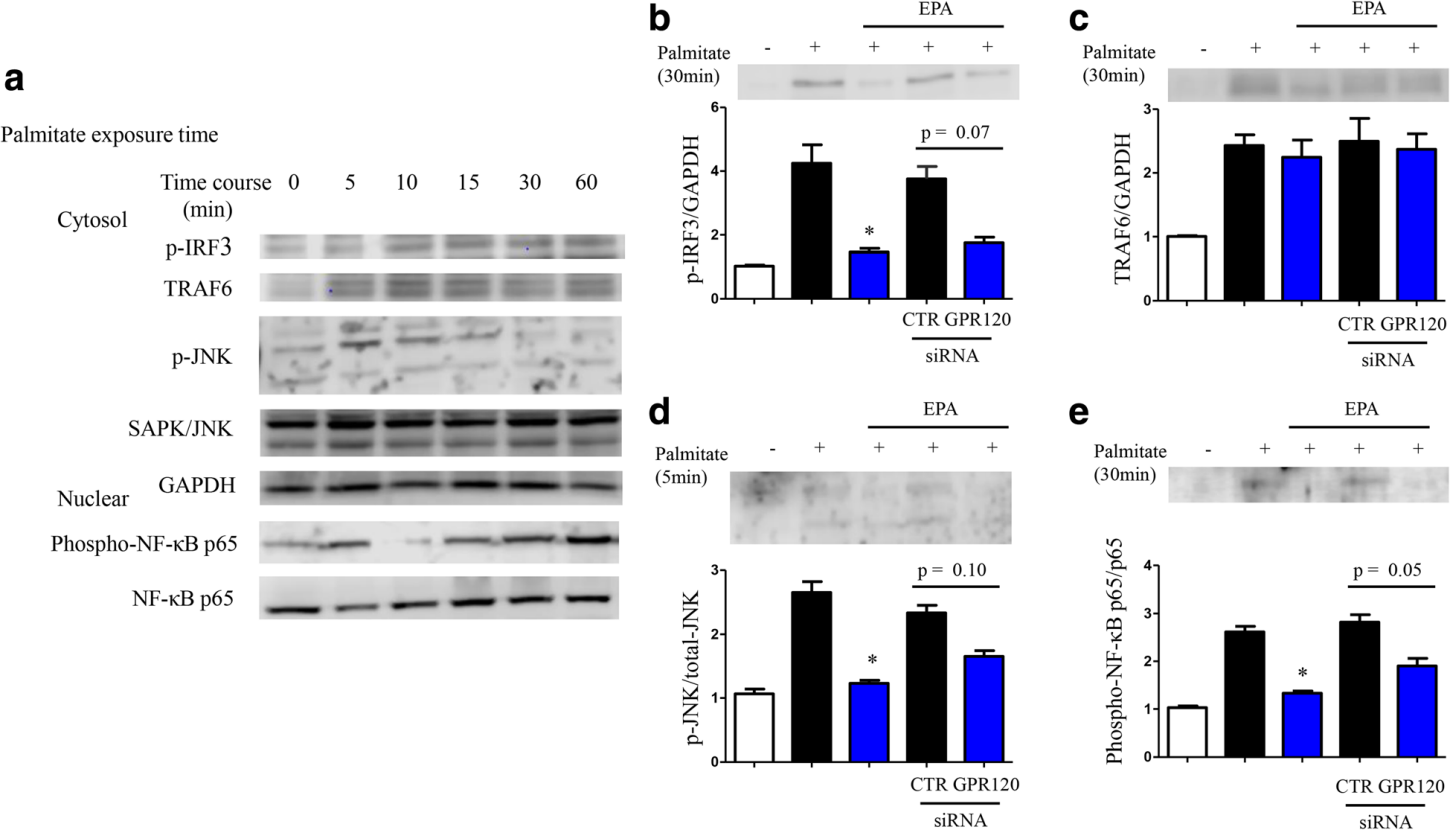
Time course of palmitate-induced expression of inflammatory molecules in 3T3-L1 adipocytes by western blot analysis (**a**). Relative protein expression levels of phosphorylated IRF3, TRAF6, phosphorylated JNK (cytosolic), and phosphorylated NF-κB p65 (nuclear) were adjusted according to control protein expression levels (GAPDH, total-JNK, and NF-κB p65; **b**–**e**). Data are presented as the mean ± SEM of three independent experiments. *P < 0.05 vs. palmitate exposure (5 or 30 min). CTR; scramble siRNA

**Fig. 3:**
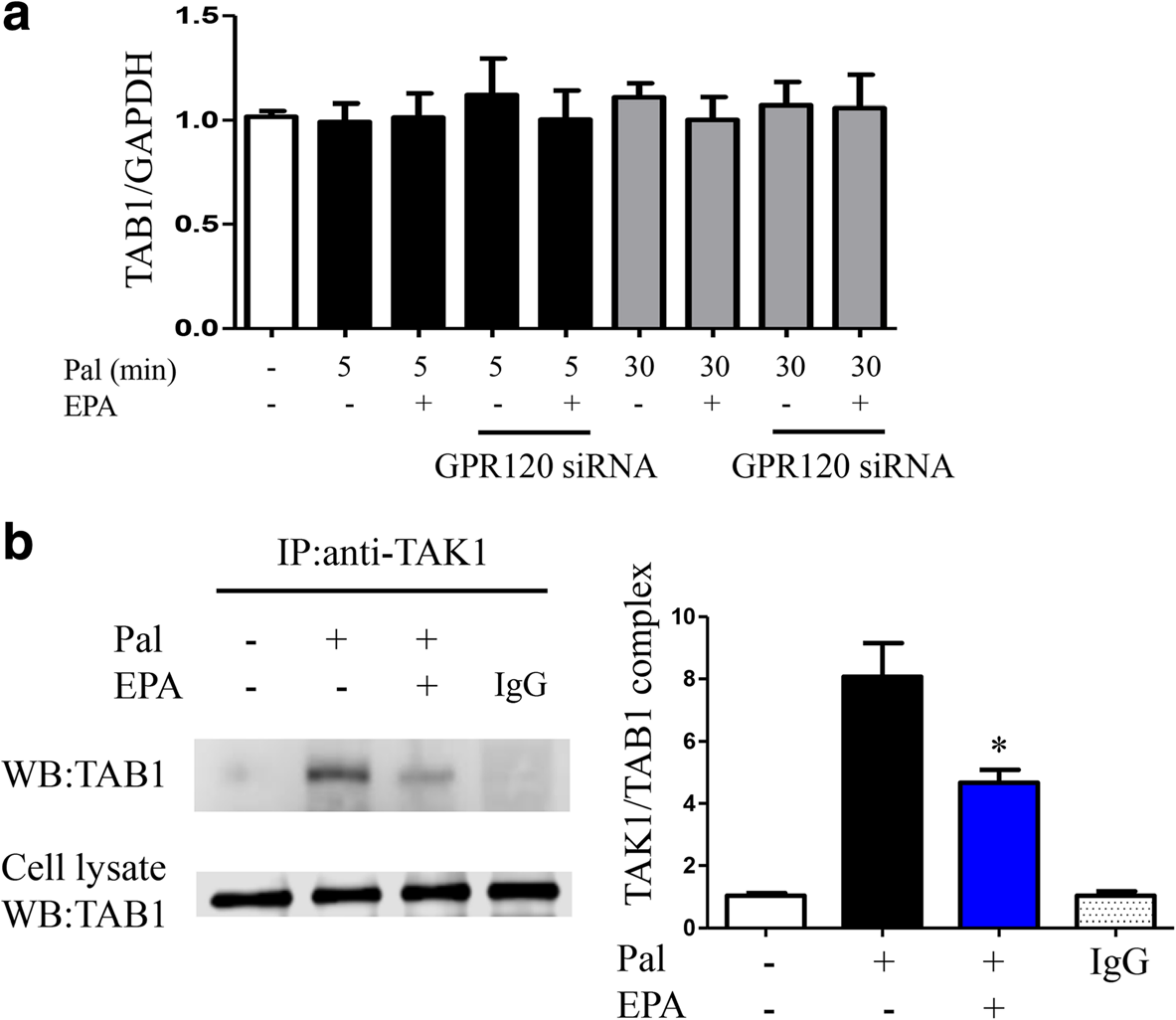
TAB-1 protein expression levels after palmitate exposure (5 or 30 min) with or without EPA pretreatment in transfected or non-transfected 3T3-L1 adipocytes (**a**). Co-immunoprecipitation analysis of TAK1 and TAB1 protein interaction (**b**). Data are presented as the mean ± SEM of three independent experiments. *P < 0.05 vs. palmitate exposure (30 min). IgG; control mouse IgG

Palmitate exposure increased monocyte chemoattractant protein-1 (MCP-1) and tumor necrosis factor (TNF)-α gene expression in 3T3-L1 adipocytes (Fig. 1). EPA pretreatment suppressed the expression of palmitate-induced inflammatory gene. Silencing of GPR120 abolished the anti-inflammatory effect of EPA; however, control scrambled siRNA did not demonstrate a significant effect (see Additional file 1: Figure S1A for silencing efficacy evaluation). GPR120 expression was not observed in pre-adipocytes, and palmitate exposure and EPA treatment had no effect on GPR120 protein expression levels (Additional file 1: Figure S1B). Palmitate induced the expression of p-IRF3, TRAF6 p-JNK, and downstream signals of TLR4 in cytosol, and phospho-NF-κB p65 in the nuclear fraction, of 3T3-L1 adipocytes (Fig. 2a). EPA pretreatment reduced p-IRF3, with a trend toward reduced p-JNK and phospho-NF-κB p65 levels. Moreover, silencing of *GPR120* tended to abolish this effect of EPA (Fig. 2b–e). Following palmitate treatment, Present or absent EPA treatment, and silencing of *GPR120* also did not affect the expression levels of TAB1 (Fig. 3a). Co-immunoprecipitation of TAK1 and TAB1 demonstrated attenuation of palmitate-induced TAK1/TAB1 complexes in response to EPA pretreatment in the cytosolic fraction (Fig. 3b). These data indicate GPR120 signaling plays an inhibitory role in palmitate-induced inflammatory signal transduction and inflammatory gene expression in adipocytes.

**Fig. 4:**
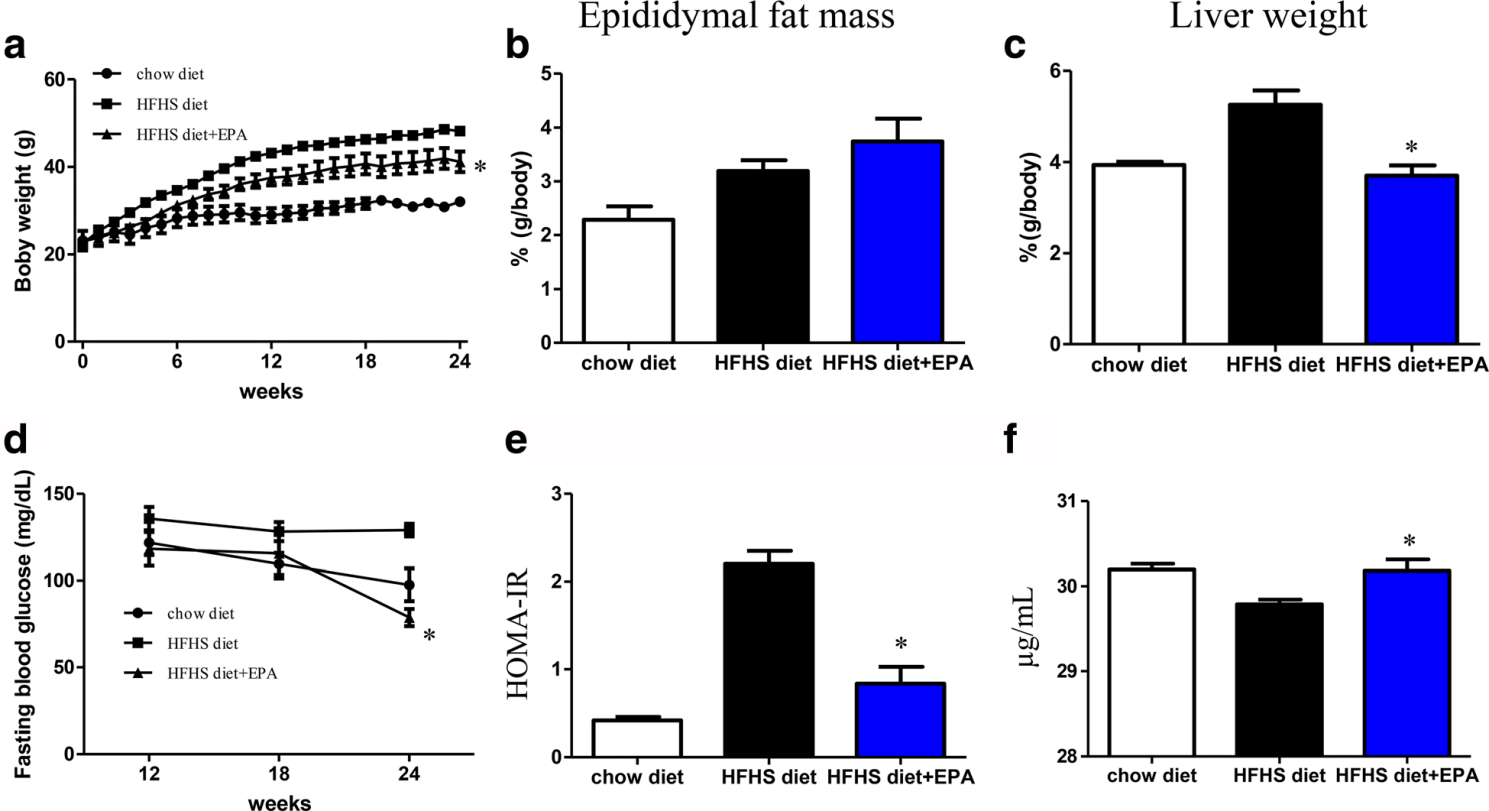
Mouse basic parameter analysis. Body weight changes (**a**), epididymal fat (**b**), and liver weight (**c**) in chow diet, high-fat/high-sucrose (HFHS) diet and HFHS diet + eicosapentaenoic acid-fed mice groups. Fasting glucose changes at 12, 18, and 24 weeks. (**d**) HOMA-IR (**e**) and serum adiponectin levels (**f**). *n* = 8–13 per group. Data are presented as means ± SEM. *P < 0.05 vs. HFHS diet group

Next, we examined the effect of EPA in HFHS diet-fed mice, an animal model of obesity and insulin resistance. Basic plasma parameters (after 6 h fasting) are shown in Table 1. EPA supplementation decreased plasma levels of total cholesterol, liver enzymes, and fasting plasma glucose levels. EPA significantly suppressed HFHS-diet induced body weight gain and reduced liver weight (%g/body), but did not affect epididymal mass (Fig. 4a–c). Fasting plasma glucose significantly reduced in the mice supplemented with HFHS containing EPA at 24 weeks (Fig. 4d). In addition, EPA supplementation improved HFHS-induced insulin resistance and increased plasma adiponectin concentration (Fig. 4e and f).

**Table 1:** Fasting plasma metabolic parameters

	chow diet (n = 10)	HFHS diet (n = 13)	HFHS diet + EPA (n = 8)
TC (mg/dL)	67 ± 3.1	181 ± 14^*^	81 ± 9.6^#^
TG (mg/dL)	28 ± 3.7	13 ± 1.6^*^	12 ± 2.0
HDL-C (mg/dL)	38 ± 2.1	63 ± 3.9^*^	50 ± 5.5
NEFA (μEq/L)	884 ± 99	630 ± 47^*^	625 ± 79
ALT (IU/L)	9.4 ± 1.9	122 ± 23^*^	32 ± 11^#^
Plasma glucose (mg/dL)	106 ± 9	126 ± 4^*^	79 ± 5^#^
Insulin (ng/mL)	1.58 ± 0.02	7.06 ± 0.41^*^	4.15 ± 0.83^#^
HbAc (%)	4.58 ± 0.05	4.51 ± 0.05	4.52 ± 0.05

Data are expressed as means ± SEM. *TC* total cholesterol, *TG* triglyceride, *HDL-C* high-density lipoprotein cholesterol, *NEFA* nonesterified fatty acid, *ALT* alanine aminotransferase, *HbA1c* glycated hemoglobin, *HFHS* high-fat/high-sucrose, *EPA* eicosapentaenoic acid ^*^ *P* < 0.05 vs. chow diet, ^#^ *P* < 0.05 vs. HFHS diet

**Fig. 5:**
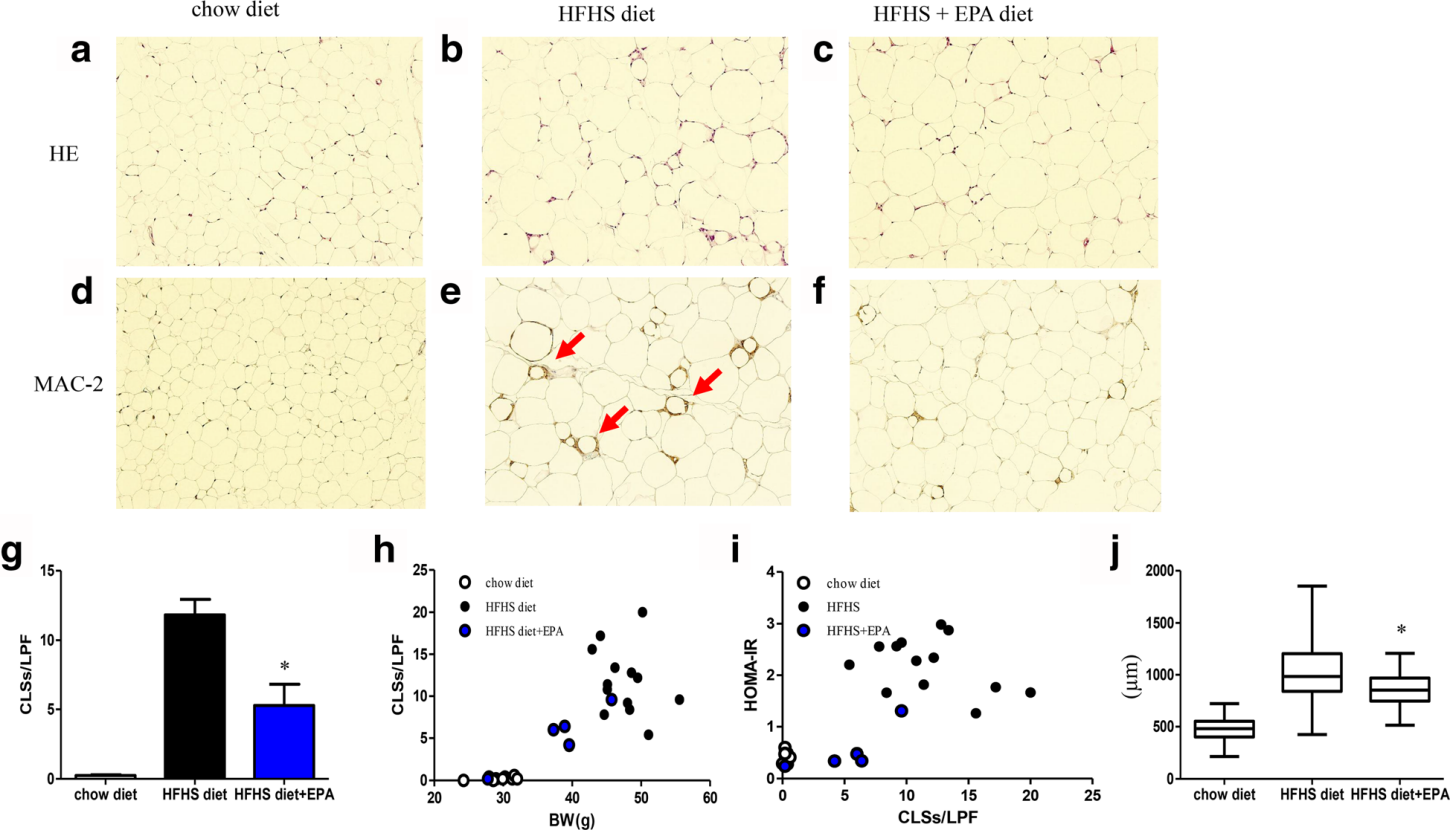
Histological analysis of mouse epididymal adipose tissue including HE staining and MAC-2 staining (**a**–**f**). Red arrows indicate CLS formation (**e**). CLS number per LPF in chow diet, high-fat/high-sucrose (HFHS) diet, and HFHS diet + eicosapentaenoic acid-fed mice groups (**g**). Correlation between body weight and CLS number (*r* = 0.80, *P* < 0.001) (**h**), and CLS number and HOMA-IR (*r* = 0.72, *P* < 0.001) (**l**). Mean adipocyte diameter in each group (**j**). *n* = 5–13 per group. Data are presented as means ± SEM. *P < 0.05 vs. HFHS diet group

Histochemical analysis demonstrated EPA supplementation inhibited the formation of CLS in edididymal fat tissue (Fig. 5a–g). Overall, body weight (BW) and CLS number (/LPF) were found to be positively correlated (r = 0.80, *P* < 0.001, Fig. 5h), and CLS number was positively correlated with HOMA-IR (*r* = 0.72, *P* < 0.001, Fig. 5i). Moreover, EPA supplementation significantly decreased HFHS diet-induced mean adipocyte diameter expansion (Fig. 5j). Liver immunohistochemistry demonstrated the presence of hepatic steatosis in mice fed an HFHS diet containing EPA (Additional file 1: Figure S2).

**Fig. 6:**
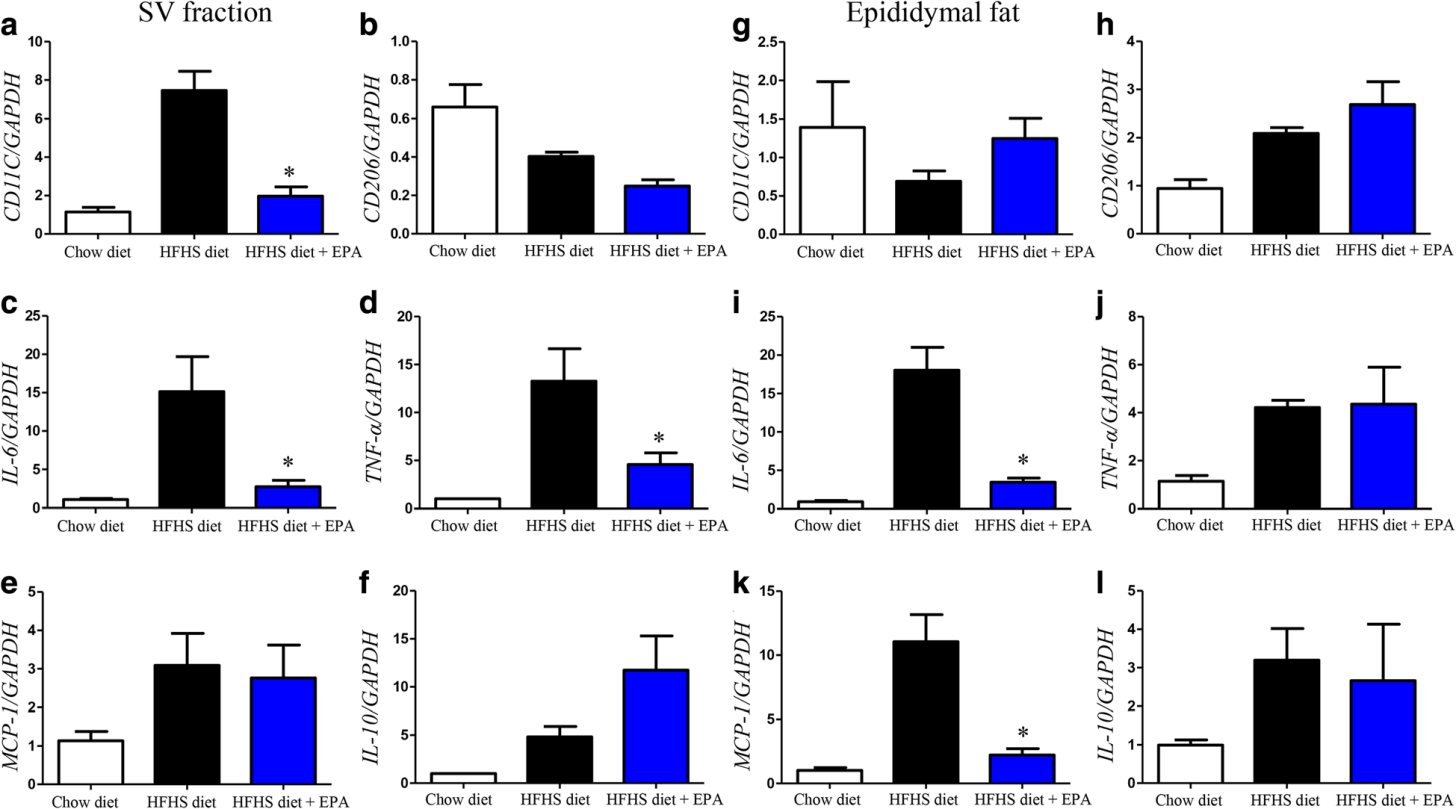
M1 (CD11c) and M2 (CD206) macrophage marker, and inflammatory (IL-6, TNF-α, MCP-1) or anti-inflammatory (IL-10) gene, expression levels and adipose tissue macrophages (ATMs) marker changes in the SV fraction (**a**–**f**) and adipose tissue (**g**–**l**). Data are presented as the mean ± SEM of five independent experiments. *P < 0.05 vs. HFHS diet group

**Fig. 7:**
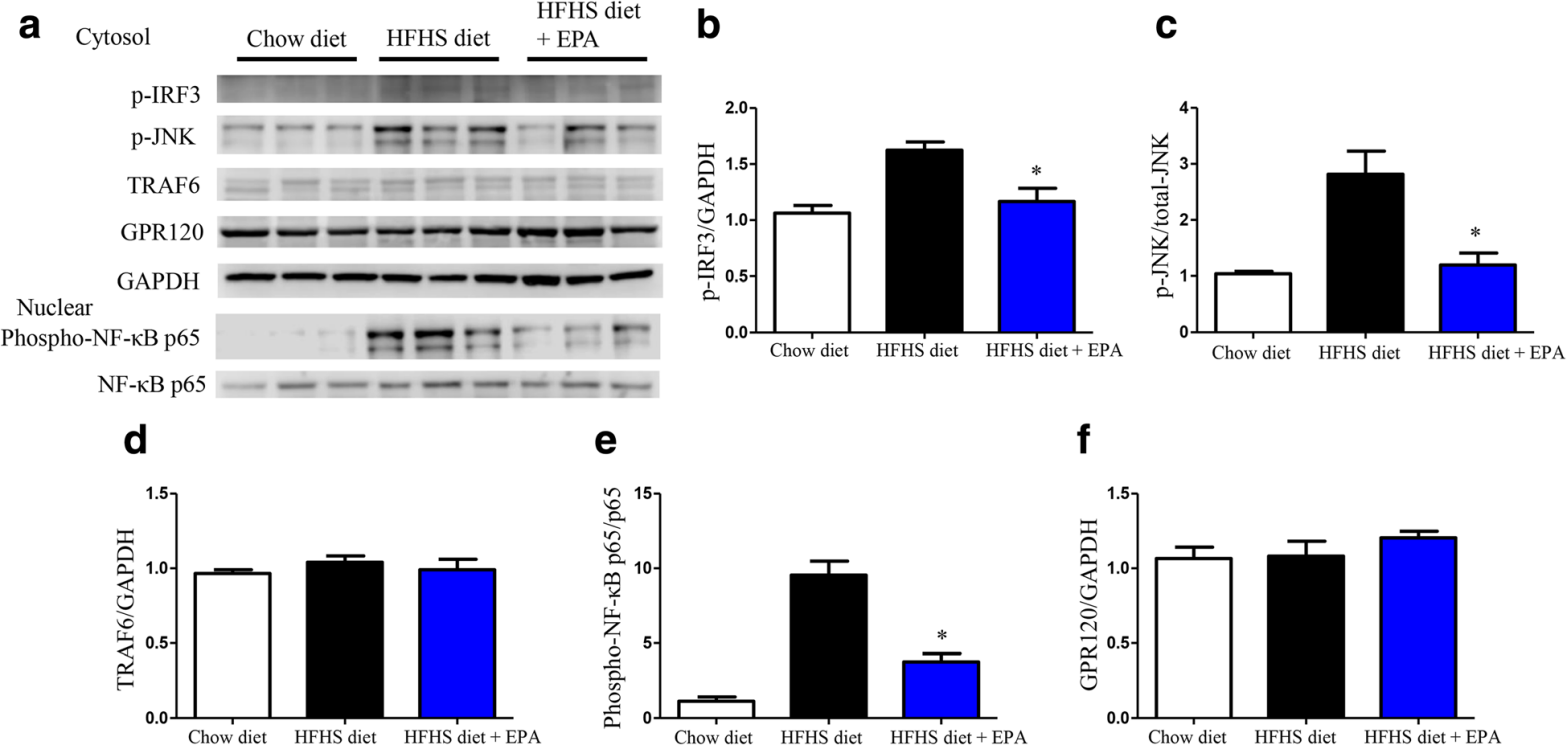
Protein expression levels of inflammatory cascade and GPR120 in epididymal adipose tissue in the three groups. **a** Relative protein expression levels of p-IRF3 (**b**), p-JNK (**c**), TRAF6 (**d**), phosphorylated NF-κB p65 (**e**) and GPR120 (**f**) in the cytosol or nuclear fraction of epididymal adipose tissue by western blot analysis. Data are presented as the mean ± SEM of three experiments. *P < 0.05 vs. HFHS diet group

We then determined inflammatory and anti-inflammatory gene expression levels, and adipose tissue macrophages (ATMs) marker changes in epididymal adipose tissues. In the SV fraction, levels of CD11c, a marker of M1 macrophage, was decreased in response to EPA supplementation. In addition, gene expression levels of IL-6 and TNF-α were also suppressed (Fig. 6a–f). In edididymal adipose tissues, the EPA-containing diet inhibited HFHS diet-induced increases in MCP-1 expression, but had no effect on CD11c and CD206, M2 macrophage markers (Fig. 6g–l). Moreover, we examined representative beige-like markers of adipocytes, uncoupling protein 1 (UCP1) and PR domain containing (PRDM) 16. We found the increased levels of mRNA of UCP1 in EPA-fed epididymal adipose tissue (Additional file 1: Figure S3). Finally, we examined inflammatory signal transduction in mouse epididymal adipose tissues. EPA supplementation suppressed HFHS diet-mediated phosphorylation of IRF3 and JNK in the cytosolic fraction. Furthermore, NF-κB p65 phosphorylation in the nuclear fraction in mice fed the HFHS diet was inhibited by EPA supplementation (Fig. 7a–e). No difference in GPR120 protein levels was observed between the three diet groups (Fig. 7f).

## Discussion

In the present study, we demonstrated that EPA suppressed palmitate-mediated expression of MCP-1 and TNF-α via GPR120 in cultured adipocytes. Moreover, EPA pretreatment reduced inflammatory signaling molecules mediated through suppression of TAK1/TAB1 complex formation. In our mouse model, EPA supplementation reduced HFHS diet-induced CLS formation, inflammatory signal transduction, and changed macrophage phenotype in adipose tissues. EPA demonstrated anti-inflammatory activity in macrophages was found to be GPR120-dependent [6]. Previous studies have reported that EPA inhibits palmitate-induced gene expression of MCP-1 or TNF-α in cultured macrophages [9, 14]. Our present results indicate GPR120 signaling in response to EPA inhibits palmitate-induced inflammation in adipocytes. Palmitate is a major SFA and induces injury in endothelial cells [15], hepatocytes [16], adipocytes [14] and pancreatic β-cells [17]. SFAs are considered a ligand of TLR4, with SFA rich-diets shown to cause low-grade chronic inflammation resulting in insulin resistance [14, 17]. It is known that TLR4 signaling activates both myeloid differentiation primary response gene (MyD) 88-depending pathway (TRAF6, JNK) and MyD88-independent pathway (IRF3) [18, 19]. In this study, we found that EPA inhibited these downstream signals transduction of TLR4, of note, GPR120 silencing tended to abolish these anti-inflammatory actions of EPA. Song et al. showed low dose palmitate (10μM) activated extracellular signal-regulated kinase (ERK)1/2, a mitogen-activated protein (MAP) kinase signaling, phosphorylation in GPR120 transfected HEK293T cells [20]. Oh et al. revealed that palmitate (100μM) also activated ERK phosphorylation in GPR120-knockdown RAW246 cells. On the other hand, the action of docosahexaenoic acid (DHA) was abolished in GPR120-knockdown RAW246 cells [6]. We considered that ω-3 fatty acids, such as DHA and EPA, show their action via GPR120, and saturated fatty acids including palmitate phosphorylates ERK independent of GPR120. In addition, our higher level of palmitate (250μM) might stimulate TNF and TLR4 signaling rather than GPR120 partial activation by palmitate. More detail studies are necessary to elucidate MAP kinase pathway signaling via GPR120.

TLR4 signal transduction also accelerates complex formation between TAK1 and TAB1, a key signal transducer of NF-κB in macrophages, and DHA inhibits TAK1/TAB1 complex formation [6]. In the present study, we demonstrate EPA has anti-inflammatory activity by inhibiting TAK1/TAB1 complex formation in 3T3-L1 adipocytes; however, protein expression of TAB1 did not change following palmitate exposure. These results indicate TAK1/TAB1 complex formation is more important than TAB1 protein expression levels. Recently, a GPR120-specific agonist was reported to reduce the interaction between TAK1 and TAB1 in intestinal epithelial cells [21]. Taken together, these results indicate GPR120-mediated anti-inflammatory pathways may represent a common system in GPR120-expressed tissues. In cultured human adipocytes, silencing of GPR120 has been shown to abolish the suppressive effect of TNF-α gene expression in response to DHA [22]. However, the results of the present study are not inconsistent with those of previous studies.

Numerous studies have reported EPA supplementation improves diet-induced fatty liver disease [23], obesity [24], and atherosclerosis [25] in mice. The results of the present study also demonstrate long-term EPA supplementation reduces BW gain, insulin resistance, and attenuates elevation of plasma alanine aminotransferase levels. CLS formation is known as a marker of the aggregation of M1 macrophages around dead or necrotic adipocytes in obese mouse models and humans [26].

As M1 macrophages are inflammatory bone marrow-derived macrophages, ATM polarity shifts from M2, a resident anti-inflammatory macrophage, to M1 macrophage phenotype in obesity. Fujisaka et al. reported that telmisartan (angiotensin II receptor blocker) or pioglitazone (Thiazolidinedione) reduces CLS formation and induces ATM polarity shifts (M1 to M2 macrophage) in diet-induced obese mice. Peroxisome proliferator-activated receptor γ (PPARγ) activation is thought to play an important role in ATM polarity shifting (M1 to M2 macrophage) as PPARγ activation in response to telmisartan and pioglitazone has been shown to reduce adipocyte size [11, 27]. In obese humans, 12-week administration of omega-3-acid ethyl esters has been shown to reduce CLS formation and MCP-1 gene expression in adipose tissue [28]. Indeed, EPA treatment increases the secretion of adiponectin by 3T3-L1 adipocytes, and plasma adiponectin levels in EPA-rich diet fed mice have been shown to be elevated [29, 30]. Chambrier et al. reported EPA treatment (50 μM, 6 h) increased mRNA levels of PPARγ in adipocytes isolated from humans [31]. We also observed a reduction in adipocyte size and increased plasma adiponectin concentrations in EPA-containing HFHS diet-fed mice. Recent study of Pinel et al. revealed that EPA supplementation inhibited fat cell hypertrophy in visceral adipose tissue and improved the adiponectin expression. Our present study was not consistent with this previous examination [32]. Song et al. reported that GPR120 stimulation promote adipogenesis and triglyceride accumulation in cultured 3T3-L1 adipocytes [33]. These newly differentiated adipocytes might act as energy store and improve glucose tolerance [34]. In recent obesity classification, metabolically healthy obese (MHO) is proposed. MHO individuals present obesity, however, have no metabolic syndrome components with increased adiponectin and reduced visceral adiposity [35]. Klöting et al. showed that visceral adipocytes size and macrophage infiltration of MHO individuals are smaller and fewer than obese metabolic syndrome individuals [36]. We did not find the differences between HFHS diet and HFHS + EPA supplementation groups, however, taken together, newly differentiated adipocytes might attenuate adipocytes size, and improve lipid metabolism and insulin resistance [34].

In addition to the above-mentioned adipogenesis aspects, it has been found that white adipose tissue could change their phenotype by some stimulation [34]. UCP1 is a key protein to regulate energy expenditure of brown adipocytes and is also expressed in beige cells, which is induced from white adipose tissue [37]. It is known that beige cells are mitochondria-rich adipocytes and thermogenic activity, then could be inducible by cold, norepinephrine, irisin and fibroblast growth factor 21 stimulations [34]. Recently, EPA or fish oil induces UCP1 and changes the phenotype of white adipocytes to beige-like adipocytes [38, 39]. We found EPA supplementation increased UCP1 mRNA expression. EPA-induced beiging of adipocytes might contribute to the improvement of metabolic profile, however, whether these actions depends on GPR120 stimulation by EPA remains unclear. More detail study is needed to elucidate this point.

However, the potential effect of EPA on adiponectin protein or gene expression in vivo remains controversial. The above study reported the administration of omega-3-acid ethyl esters had no effect on adipocyte size or plasma adiponectin concentrations in a population of obese individuals [28]. These differences may be induced by EPA in vivo. In animal studies, dietary EPA doses are typically 5% (wt/wt) or more. However, the administration of doses at these levels in daily dietary forms is clinically challenging. Accordingly, comparisons between animal and human studies should be interpreted with caution, especially among nutrient supplementation studies.

Otherwise, improved insulin resistance may be an effect of decreased MCP-1 gene expression in adipose tissue, thereby reducing the involvement of M1 macrophage in adipose tissue. HFHS diet-induced adipose inflammation may therefore be improved. We demonstrated EPA supplementation reduces expression of the M1 macrophage marker, CD11c, in the SV fraction. In fact, several in vitro and in vivo studies have reported that EPA-treatment inhibits palmitate-induced MCP-1 induction in cultured adipocytes [14] and HFHS diet-induced MCP-1 induction in adipose tissues [24]. A direct effect of EPA on adipocytes may contribute to anti-inflammatory action via suppression of MCP-1 gene expression in adipose tissues. Oh et al. demonstrated that ω-3 fatty acid supplementation has no effect in macrophage specific GPR120 knock-down mice [6]. However, whether the effect of ATM polarity shift depends on macrophage or adipose GPR120 has yet to be elucidated.

In the present study, EPA inhibited HFHS diet-induced inflammation. Sato et al. reported similar results, with 20 weeks of EPA supplementation found to suppress BW gain and MCP-1 gene expression in HFHS diet-fed mice, but to have no effect in high-fat (HF) diet-fed mice [24].

Sucrose is an independent risk factor for metabolic syndrome and non-alcoholic fatty liver disease (NAFLD) [40, 41]. We also observed that EPA supplementation inhibits HFHS-mediated steatosis and CLS formation in mouse liver (Additional file 1: Figure S2), and decreases the lipid fraction of LDL (Additional file 1: Figure S4 and Table S2). HFHS diet has been shown to induce more severe hepatic triglyceride accumulation and hepatic lipogenesis [24]. In addition, oral administration of EPA affects systemic metabolism. Further studies are required to elucidate the organ-specific agonistic effect of GPR120.

## Conclusions

In the present study, we demonstrate EPA treatment inhibits TAK1/TAB1 complex formation and inflammatory responses via GPR120 in cultured adipocytes. In addition, EPA supplementation reduced HFHS diet-induced CLS formation and MCP-1 gene expression in mouse adipose tissues. These results indicate adipose GPR120 plays an important role in anti-inflammatory activity.

## Additional file

Additional file 1: Figure S1. Efficacy of siRNA for GPR120 (a) and GPR120 protein expression analysis in 3T3-L1 adipocytes (CTR, eicosapentaenoic acid 6h, palmitate 24h exposure) and preadipocytes by western bot (b). **Figure S2.** Liver histological analysis by HE staining and MAC-2 staining (×100). Red arrows indicated hepatic CLS formation. **Figure S3.** EPA supplementation-induced beige-like marker in epididymal adipose tissue. mRNA expression of uncoupling protein 1 (UCP1) and PR domain containing (PRDM) 16 was evaluated in each diet groups. Data are presented as the mean ± SEM of five independent experiments. **Figure S4.** Cholesterol and triglyceride profile by HPLC. Lipid fraction of chow diet (a), high-fat/high-sucrose (HFHS) diet (b), and HFHS diet + eicosapentaenoic acid (c). The blue line represents triglyceride and pink line represents cholesterol contents. The LDL peak was found in HFHS diet plasma (b). VLDL, very low-density lipoprotein; LDL, low-density lipoprotein; HDL, high-density lipoprotein; FG, free glycerol. Method of this analysis is described in the following supplementary method. **Table S1.** Taq Man® Gene Expression ID information. **Table S2.** Analysis of plasma lipoproteins by HPLC. (PDF 497 kb)

## Abbreviations

ATMs: Adipose tissue macrophages
CLSs: Crown-like structures
DHA: Docosanexaenoic acid
EPA: Eicosapentaenoic acid
FFAs: Free fatty acids
GPR120: G-protein coupled receptor 120
HFHS: High-fat/high-sucrose
HOMA-IR: Homeostasis model for insulin resistance
IRF3: Interferon regulatory factor 3
JNK: C-Jun N-terminal kinase
LPS: Lipopolysaccharide
MCP-1: Monocyte chemoattractant protein-1
NF-κB: Nuclear Factor-κB
PPARγ: Peroxisome proliferator-activated receptor γ
PUFAs: polyunsaturated free fatty acids
SFAs: saturated free fatty acids
TAB1: TGF-β activated kinase 1 binding protein 1
TAK1: TGF-β activated kinase 1
TGF-β: tumor growth factor β
TLR4: toll-like receptor 4
TNF-α: tumor necrosis factor-α
TRAF6: TNF receptor associated factor 6
