# Supplementary material for: Eicosapentaenoic acid shows anti-inflammatory effect via GPR120 in 3T3-L1 adipocytes and attenuates adipose tissue inflammation in diet-induced obese mice
Source: Nutr Metab (Lond). 2017 May 8;14:33. doi: 10.1186/s12986-017-0188-0 (PMC5422876; doi:10.1186/s12986-017-0188-0)
Supplement: Additional file 1: Figure S1. — Efficacy of siRNA for GPR120 (a) and GPR120 protein expression analysis in 3T3-L1 adipocytes (CTR, eicosapentaenoic acid 6h, palmitate 24h exposure) and preadipocytes by western bot (b). Figure S2. Liver histological analysis by HE staining and MAC-2 staining (×100). Red arrows indicated hepatic CLS formation. Figure S3. EPA supplementation-induced beige-like marker in epididymal adipose tissue. mRNA expression of uncoupling protein 1 (UCP1) and PR domain containing (PRDM) 16 was evaluated in each diet groups. Data are presented as the mean ± SEM of five independent experiments. Figure S4. Cholesterol and triglyceride profile by HPLC. Lipid fraction of chow diet (a), high-fat/high-sucrose (HFHS) diet (b), and HFHS diet + eicosapentaenoic acid (c). The blue line represents triglyceride and pink line represents cholesterol contents. The LDL peak was found in HFHS diet plasma (b). VLDL, very low-density lipoprotein; LDL, low-density lipoprotein; HDL, high-density lipoprotein; FG, free glycerol. Method of this analysis is described in the following supplementary method. Table S1. Taq Man® Gene Expression ID information. Table S2. Analysis of plasma lipoproteins by HPLC. (PDF 497 kb) [file 12986_2017_188_MOESM1_ESM.pdf]

Supplementary Material

Supplementary Figure 1.

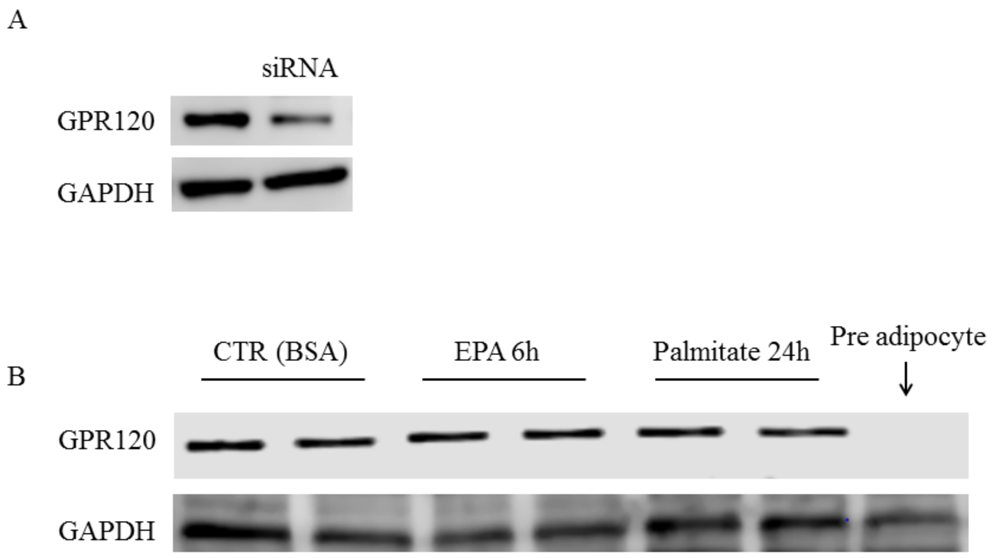

Supplementary Figure 1. Efficacy of siRNA for GPR120 (a) and GPR120 protein expression analysis in 3T3-L1 adipocytes (CTR, eicosapentaenoic acid 6h, palmitate 24h exposure) and preadipocytes by western bot (b).

Supplementary Figure 2.

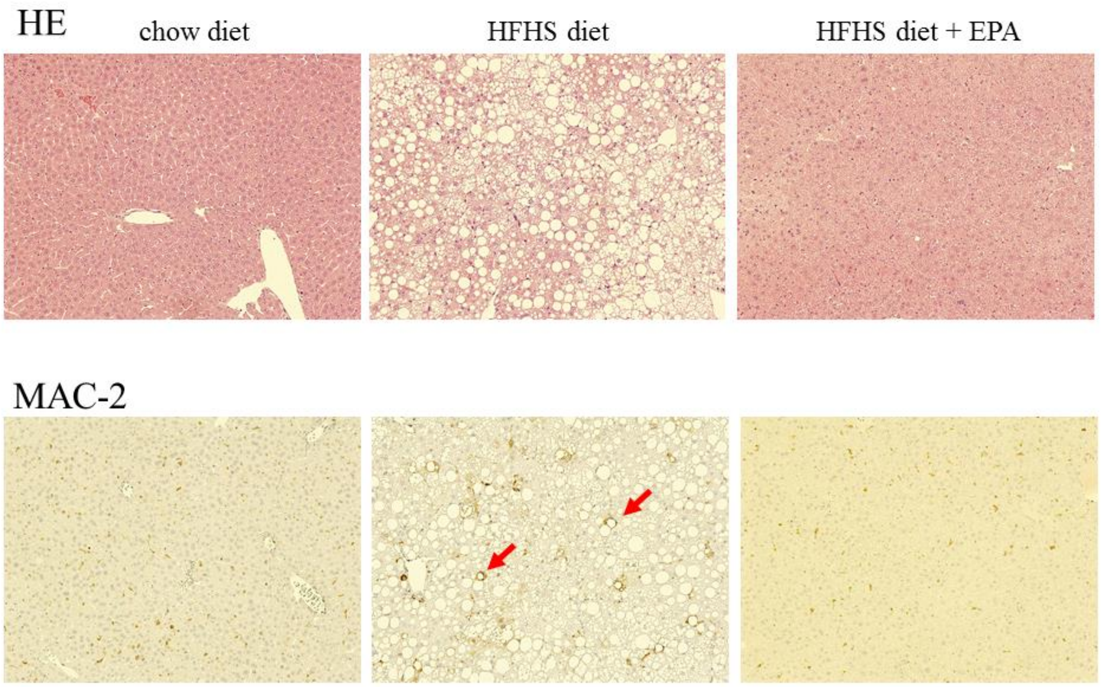

Supplementary Figure 2. Liver histological analysis by HE staining and MAC-2 staining (×100). Red arrows indicated hepatic CLS formation.

Supplementary Figure 3.

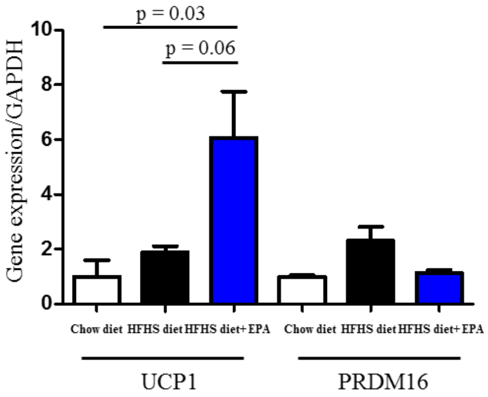

Supplementary Figure 3. EPA supplementation-induced beige-like marker in epididymal adipose tissue. mRNA expression of uncoupling protein 1 (UCP1) and PR domain containing (PRDM) 16 was evaluated in each diet groups. Data are presented as the mean ± SEM of five independent experiments.

Supplementary Figure 4.

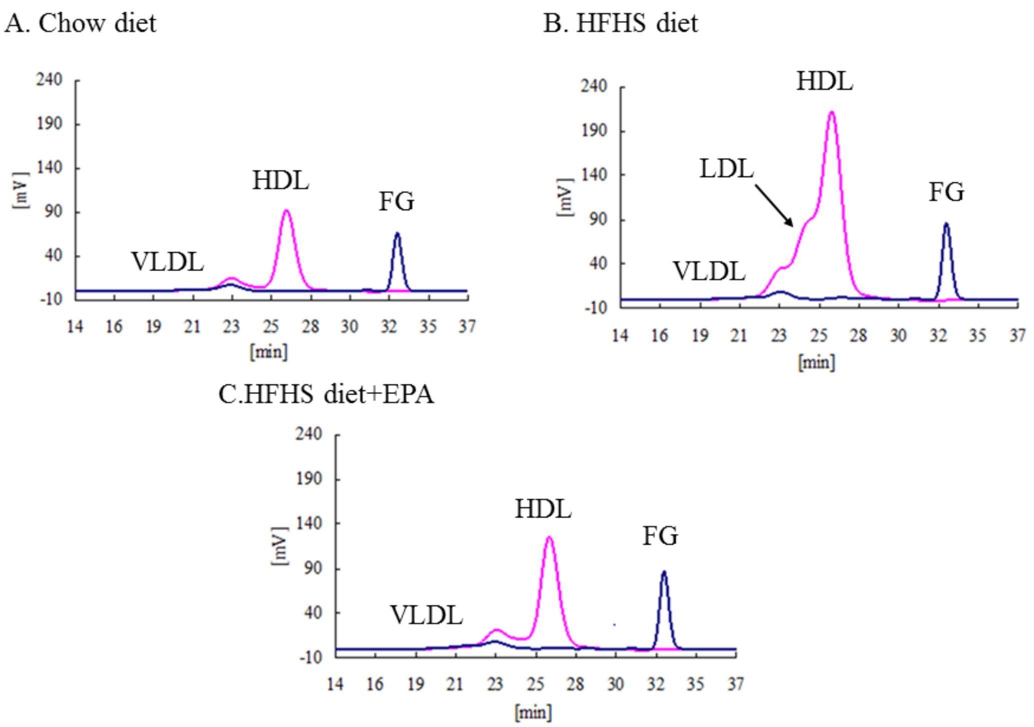

Supplementary Figure 4. Cholesterol and triglyceride profile by HPLC.

Lipid fraction of chow diet (a), high-fat/high-sucrose (HFHS) diet (b), and HFHS diet + eicosapentaenoic acid (c). The blue line represents triglyceride and pink line represents cholesterol contents. The LDL peak was found in HFHS diet plasma (b). VLDL, very low-density lipoprotein; LDL, low-density lipoprotein; HDL, high-density lipoprotein; FG, free glycerol. Method of this analysis is described in the following supplementary method.

Supplementary Table 1. Taq Man® Gene Expression ID information

Taq Man Gene	Assay ID
MCP-1	Mm00441242_m1
TNF-α	Mm00443258_m1
CD11c	Mm00498701_m1
CD206	Mm01329362_m1
IL-6	Mm00446190_m1
IL-10	Mm01288386_m1
UCP1	Mm01244861_m1
PRDM16	Mm00712556_m1
GAPDH	Mm99999915_g1

Supplementary Table 2. Analysis of plasma lipoproteins by HPLC

		Cholesterol (mg/dL)				Triglyceride (mg/dL)			
**Group**	**Weeks**	**CM**	**VLDL**	**LDL**	**HDL**	**CM**	**VLDL**	**LDL**	**HDL**
	12 w	0.7	5.68	15.92	72.46	2.45	18.11	10.81	1.77
Chow diet	18 w	0.78	6.88	15.2	70.51	4.81	27.95	11.53	2.38
	24 w	0.48	3.4	9.39	46.78	1.69	8.41	10.93	1.64
	12 w	0.3	2.37	45.02	129.5	0.8	5.94	10.76	2.59
HFHS diet	18 w	0.23	3.23	51.19	128.9	0.46	4.16	12.01	3.98
	24 w	0.16	3.87	55.07	128.8	0.53	7.81	13.53	4.6
	12 w	0.11	2.6	12.94	68.5	0.21	6.93	10.08	2.83
HFHS diet + EPA	18 w	0.16	2.63	17.47	67.23	0.28	5.28	9.22	3.37
	24 w	0.17	3.59	15.92	66.32	0.53	11.45	12.99	3.2

CM, chylomicron; VLDL, very low-density lipoprotein; LDL, low-density lipoprotein; HDL, high-density lipoprotein. CM (>80 nm), VLDL (30–80 nm), LDL (16–30 nm), HDL (8–16 nm); EPA, eicosapentaenoic acid.

Supplementary method

Plasma lipoprotein fractions were analyzed with a high-performance liquid chromatography (HPLC) system at Skylight Biotech (LipoSEARCH, Akita, Japan). For this analysis, plasma samples were collected from each group and pooled. Results are shown in Supplementary Table 2.
